# Autistic differences in the temporal dynamics of social attention

**DOI:** 10.1177/1362361321998573

**Published:** 2021-03-11

**Authors:** Nicholas Hedger, Bhismadev Chakrabarti

**Affiliations:** Centre for Autism, School of Psychology & Clinical Language Sciences, University of Reading, UK

**Keywords:** attention, autism, eye-tracking, gaze, social attention, temporal modelling

## Abstract

Individuals with autism spectrum disorders typically exhibit reduced visual attention towards social stimuli relative to neurotypical individuals. Importantly, however, attention is not a static process, and it remains unclear how such effects may manifest over time. Exploring these momentary changes in gaze behaviour can more clearly illustrate how individuals respond to social stimuli and provide insight into the mechanisms underlying reduced social attention in autism spectrum disorder. Using a simple passive eye-tracking task with competing presentations of social and nonsocial stimuli, we examine the different ways in which attention to social stimuli evolves over time in neurotypical adults and adults with and autism spectrum disorders. Our temporal modelling of gaze behaviour revealed divergent temporal profiles of social attention in neurotypical and observers with autism. Neurotypical data showed an initial increase in social attention, a ‘decay’ and subsequent ‘recovery’ after prolonged viewing. By contrast, in individuals with autism spectrum disorder, social attention decayed over time in a linear fashion without recovery after prolonged viewing. We speculate that the ‘gaze cascade’ effect that maintains selection of social stimuli in neurotypical observers is disrupted in individuals with high autistic traits. Considering these temporal components of gaze behaviour may enhance behavioural phenotypes and theories of social attention in autism spectrum disorder.

**Lay abstract:**

One behaviour often observed in individuals with autism is that they tend to look less towards social stimuli relative to neurotypical individuals. For instance, many eye-tracking studies have shown that individuals with autism will look less towards people and more towards objects in scenes. However, we currently know very little about how these behaviours change over time. Tracking these moment-to-moment changes in looking behaviour in individuals with autism can more clearly illustrate how they respond to social stimuli. In this study, adults with and without autism were presented with displays of social and non-social stimuli, while looking behaviours were measured by eye-tracking. We found large differences in how the two groups looked towards social stimuli over time. Neurotypical individuals initially showed a high probability of looking towards social stimuli, then a decline in probability, and a subsequent increase in probability after prolonged viewing. By contrast, individuals with autism showed an initial increase in probability, followed by a continuous decline in probability that did not recover. This pattern of results may indicate that individuals with autism exhibit reduced responsivity to the reward value of social stimuli. Moreover, our data suggest that exploring the temporal nature of gaze behaviours can lead to more precise explanatory theories of attention in autism.

It has been argued that the core clinical symptoms of autism result from differences in how people with autism attend to the world during infancy and childhood (Dawson et al., 2004; Toth et al., 2006). The literature is replete with evidence that individuals with autism spectrum disorders (ASD)^1^ exhibit reduced attention to social stimuli relative to the neurotypical (NT) population. These observations have emanated primarily from eye-tracking paradigms, wherein observers are presented with competing displays of social and nonsocial stimuli (Chita-Tegmark, 2016; Hedger et al., 2020). For instance, when presented with competing animations of upright and inverted human biological motion, NT children gaze for longer at upright animations that resemble human motion, whereas this preference is not observed in children with ASD, who instead displayed preferences for nonsocial physical contingencies (Klin et al., 2009). Similarly, when presented with competing videos of social interactions and geometric patterns, NT individuals gaze for longer at the social interactions, whereas this tendency is reduced, or reversed in individuals with ASD (Pierce et al., 2016). Meta-analyses indicate that this reduced social attention in ASD is a robust effect that generalises across a number of stimulus conditions and across child and adult samples (Chita-Tegmark, 2016; Frazier et al., 2017). Such eye-tracking tasks are simple to administer and do not require advanced motor responses or language abilities. As such, there is widespread enthusiasm for the idea that eye-tracking paradigms may provide practical, inexpensive and scalable tools for screening for ASD risk, particularly in low-resource settings (Falck-Ytter et al., 2013; Sasson & Elison, 2012).

One important thing to consider about such studies is that social gaze behaviour is typically described by summary metrics such as the average proportion of time that gaze is directed to social stimuli. Figure 1 (upper row) depicts an example of such gaze proportion data for 3 separate observers from the present study. When viewing this aggregated form of the data, all 3 observers appear to exhibit similar behaviour, spending an average of ~ 50% of the trial directing their gaze towards the social images. However, if we instead represent their data as an average time series (Figure 1, lower row), this reveals that the three observers have patterns of gaze behaviour that evolve in dramatically different ways. The first observer exhibits an initial preference for the nonsocial images, then the social, before returning to the nonsocial. The second observer exhibits an initial bias for social stimuli, but this bias decays over time. The final observer displays more complex switching behaviour. These examples demonstrate an important caveat of working with time-aggregated data: the same mean gaze proportions could be the product of a large and diverse set of gaze proportion timeseries. As such, by failing to consider the temporal domain, important differences in gaze behaviour can remain undetected.

**Figure 1.**
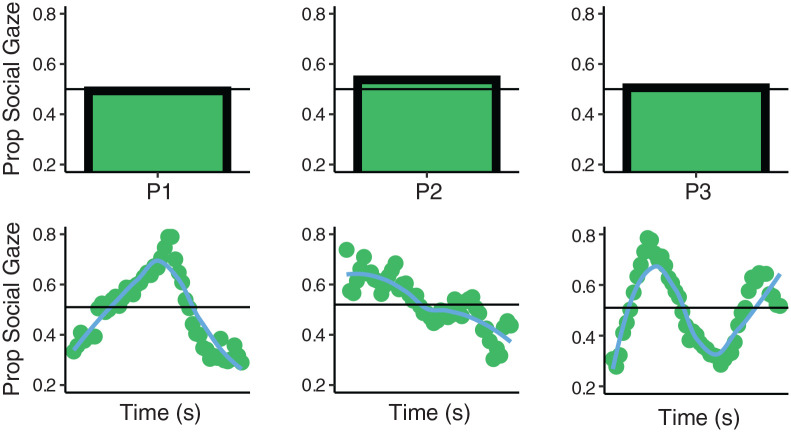
Shows gaze proportion data for 3 participants from the present study who viewed competing displays of social and nonsocial images for 5 s. The upper row shows the average gaze proportion data. Horizontal line demarcates 50% of trial time spent looking at social image. The lower row shows average time-series data for the same 3 observers. Points indicate gaze proportion within 100 ms time bins from the start to end of trial. Line indicates local weighted regression fit to the data.

Recent work has demonstrated the advantages of considering these temporal aspects of social attention. Tracking the moment-by-moment changes in eye-movement behaviour can illustrate more vividly the differences in how individuals respond to social stimuli. Critically, treating the data as a timeseries allows us to determine *when* differences in social attention tend to arise between individuals and groups and relate these to social trait characteristics of the observer. Specifically, in a NT adult sample, it has been found that individuals with high empathy showed an increased bias for social images, particularly after prolonged viewing (Hedger et al., 2018). Another study that investigated the temporal aspects of social attention was conducted by Constantino and colleagues (Constantino et al., 2017). The authors observed that when viewing social scenes, monozygotic twins were more likely to exhibit co-occurring saccadic eye movements than dizygotic twins, indicating a genetic influence on the timing of eye-movement behaviours.

Considering these observations together, there is evidence that social trait characteristics such as empathy can predict different temporal profiles of social attention. Moreover, there is evidence for a genetic influence on the timing of social attention behaviours. ASDs, which are marked by both (a) a large genetic component (Abrahams & Geschwind, 2008; de la Torre-Ubieta et al., 2016) and (b) atypicalities in some empathy-related processes (Jones et al., 2010), are associated with reduced social attention. However, currently, we know very little about the temporal features of gaze behaviour that underlie this reduction. This link between empathy and autism, taken together with the observations of Hedger et al. (2018), generates the prediction that the reduction in social attention observed in ASD may become reliable several seconds after stimulus presentation and that this will be driven by an increased tendency for NT individuals to return gaze to social images. Therefore, the aims of the present study were to (a) establish *when* autistic differences in social attention become robust and (b) relate these group differences to individual variation in autism-related traits (c) provide an explicit model that describes how social attention tends to evolve over time in observers with ASD and NT observers.

## Methods

### Participants

Participant characteristics are summarised in Table 1. Our NT group was formed of 30 students and academic staff recruited from the University of Reading Campus. Our ASD group were recruited through the Centre for Autism database of research volunteers. Volunteers were not considered for recruitment if they were under 18 years of age, or if their database record indicated concurrent diagnosis of attention deficit hyperactivity disorder (ADHD), a learning disorder, conduct disorder, Tourette’s syndrome or epilepsy. With these restrictions applied, the ASD sample was formed of 23 adults with a *Diagnostic and Statistical Manual of Mental Disorders* (4th ed., text rev; *DSM*-IV TR; American Psychiatric Association, 2013) based diagnosis of ASD from a recognised clinic. Note that this sample of participants is the same as those reported in (Hedger & Chakrabarti, 2021). All participants had normal/ corrected to normal vision. There were no significant differences between groups in terms of either age (*t*(51) = −1.09, *p* = 0.277), or gender (*χ*^2^(1) = .25, *p* = 0.615). The majority of participants identified as ‘White’ (NT: 93.3%, ASD: 82.6%). The remainder identified themselves as belonging to ‘more than one race’ (NT: 6.7%, ASD = 8.7%), or preferred not to disclose racial information (ASD: 8.7%). Specific data on socioeconomic status of the sample were not recorded. Ethical clearance was obtained from the University of Reading Ethics committee (approval code: 2014-059-BC), and all participants gave fully informed consent.

**Table 1.** Summary of Participant Demographics.

Group	*N*	*AQ (M*,)	Age *(M, SD)*	Gender (*Male, Female*)
NT	30	16.60 (7.70)	33.3 (13.74)	11,19
ASD	23	38.13 (5.85)	37.34 (12.64)	10,13

AQ: Autism Spectrum Quotient; *SD*: standard deviation; NT: neurotypical; ASD: autism spectrum disorders.

One plausible concern could be that group differences in social attention may be modulated by reduced cognitive functioning and not differences in autistic traits per se. However, the currently available data indicate reduced social attention in ASD is orthogonal to IQ. For instance, two large-scale meta analyses indicate that social attention differences are not modulated by verbal or nonverbal IQ matching of the NT and ASD groups (Chita-Tegmark, 2016; Hedger et al., 2020) and remain stable across IQ differences between groups (Frazier et al., 2017). Moreover, numerous studies with developmentally delayed (DD) control groups show reduced social attention in ASD relative to *both* DD and NT subjects (Chawarska et al., 2012; Klin et al., 2009; Shic et al., 2011). To provide a measure of cognitive ability of the ASD group, all ASD participants completed the Wechsler Abbreviated Scale of Intelligence (WASI), which estimates the participant’s cognitive ability as a percentile of the general population (Wechsler, 2008). The mean WASI of the ASD group was 115 (*SD* = 10.53), indicating a high level of functioning and cognitive ability comparable to the university population (Lassiter et al., 2001; Wilson et al., 2014). To provide a measure of autism-related traits, all participants completed the Autism Spectrum Quotient (AQ: Baron-Cohen et al., 2001). A robust difference was detected between the NT and ASD group in AQ scores (*t*(51) = −11.56, *p* < 0.001). The ASD group had substantially higher AQ (*d* = 3.23 (2.38, 4.08)) than the NT group. This effect size is consistent with a 99% chance that a randomly sampled observer from the ASD group will have a higher AQ score than a randomly sampled observer from the NT Group (Lakens, 2013). The mean AQ scores for the groups were either side of 26, which is considered to be a cutoff with good screening properties and discriminative validity (Woodbury-Smith et al., 2005).^2^

### Stimuli

**Figure 2.**
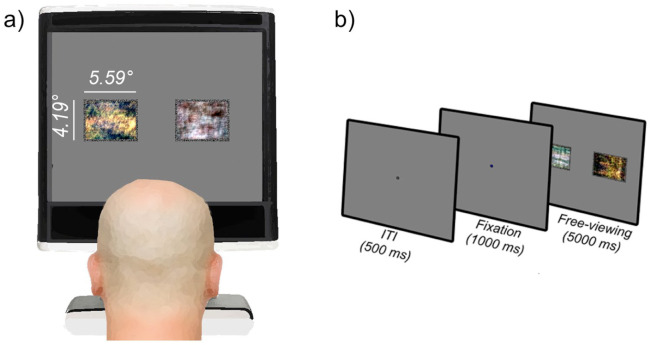
(a) Schematic of experimental setup. Two phase-scrambled images are shown. (b) Schematic of trial sequence.

We employed 30 pairs of social and nonsocial reward images, which were the same as those used in (Chakrabarti et al., 2017). Images were recovered from the international Affective Picture System (Lang et al., 2008) and publicly available creative common licenced databases. Social images were scenes involving happy groups of people, whereas nonsocial images involved food, natural scenery and money (see Supplementary Material S1 for further details of selection criteria). Participants were seated 60 cm from a 1280 × 1024 pixel resolution monitor and gaze was recorded via a Tobii T60 eyetracker sampling at 60 hz. The resolution of the display was 41.29 pixels/degree of visual angle (DVA). Stimuli subtended 5.59 × 4.19 DVA. To minimise the influence of extraneous sensory and affective differences between image pairs, all pairs were matched as closely as possible in terms of low-level properties (luminance, contrast, Koch saliency) as well as perceived valence and arousal (see Supplementary Material S1). In addition, to further characterise the influence of low-level confounds, we presented two *stimulus types.* One set of images were *intact*, and another set of images were phase scrambled. This manipulation maintains the mean luminance, contrast, spatial frequency and colour profile of the intact images, but renders them unrecognisable (Figure 2(a)). The logic of this manipulation is that if simple low-level variability between image pairs drives a bias towards social images, we would expect to find a social bias of the same magnitude in the intact and scrambled condition. All stimuli were presented using MATLAB with Psychtoolbox extensions (Brainard, 1997).

### Procedure

At the start of the experiment, all observers completed a 9 point calibration and subsequent 9 point validation procedure. This procedure was repeated if necessary, until validation performance indicated < 1DVA spatial accuracy and < .20 DVA precision in the validation samples. Subsequently, participants completed the free-viewing task: Observers were informed that they would be presented with pairs of images side by side for 5000 ms, and the only instruction given to the observers was to ‘take a good look at the images’. Figure 2(b) depicts the trial sequence: observers were presented with a fixation cross for 1000 ms, followed by a pair of social and non-social stimuli that were presented equidistant (4.6 DVA) from the position of the fixation cross for 5000 ms. Observers completed 60 trials in total (30 image pairs, 2 stimulus types). Each participant was presented with the same pseudo-random trial order, wherein an equal number of social and nonsocial stimuli were presented either side of fixation.

## Results

### Data reduction and analytic approach

The raw data supporting this article are publicly available via the ‘Figshare’ repository (Hedger, 2020). Using Grafix Fixations Coder software (Saez de Urabain et al., 2015), we combined raw gaze coordinates from the left and right eyes into a single set of X and Y coordinates and smoothed this time-series by submitting it to bilateral filtering algorithm (Durand & Dorsey, 2002). Finally, we interpolated missing portions of data that were briefer than 150 ms. The cutoff of 150 ms was based on previous literature that indicates saccadic programming takes around 130 ms and so this interpolations of less than 150 ms should prevent interpolating an entire saccade-fixation-saccade sequence (Wass et al., 2013). We removed trials for which gaze failed to record for more than 60% of the trial (3.89% of the data). There were no differences between ASD and NT groups in terms of the amount of data removed (*t*(51) = −.03, *p* = 0.974). Unless specified otherwise, we fit generalised linear mixed effects models to evaluate hypotheses. Each fixed effect that was evaluated was entered into the model with a corresponding by-subject random slope (Barr et al., 2013). Reported *p* values were obtained by likelihood ratio tests that compare models with the coefficients to those without them (recommended by Barr et al., 2013).

### Aggregated social bias

Data reduction was performed via the ‘eyetrackingR’ package, implemented in the R programming language (Dink & Ferguson, 2015). The display coordinates occupied by the social and non-social images on each trial were defined as areas of interest (AOIs). To evaluate the global tendency to attend to social images, we first analysed the data by aggregating across the time dimension. To this end, we reduced the raw gaze data for each participant into the proportion of trial time that gaze was directed into the social AOI and non-social AOI (Note that the computation of these proportions ignored rare instances where the participants gaze was directed into neither AOI (.63% of the data)).

**Figure 3.**
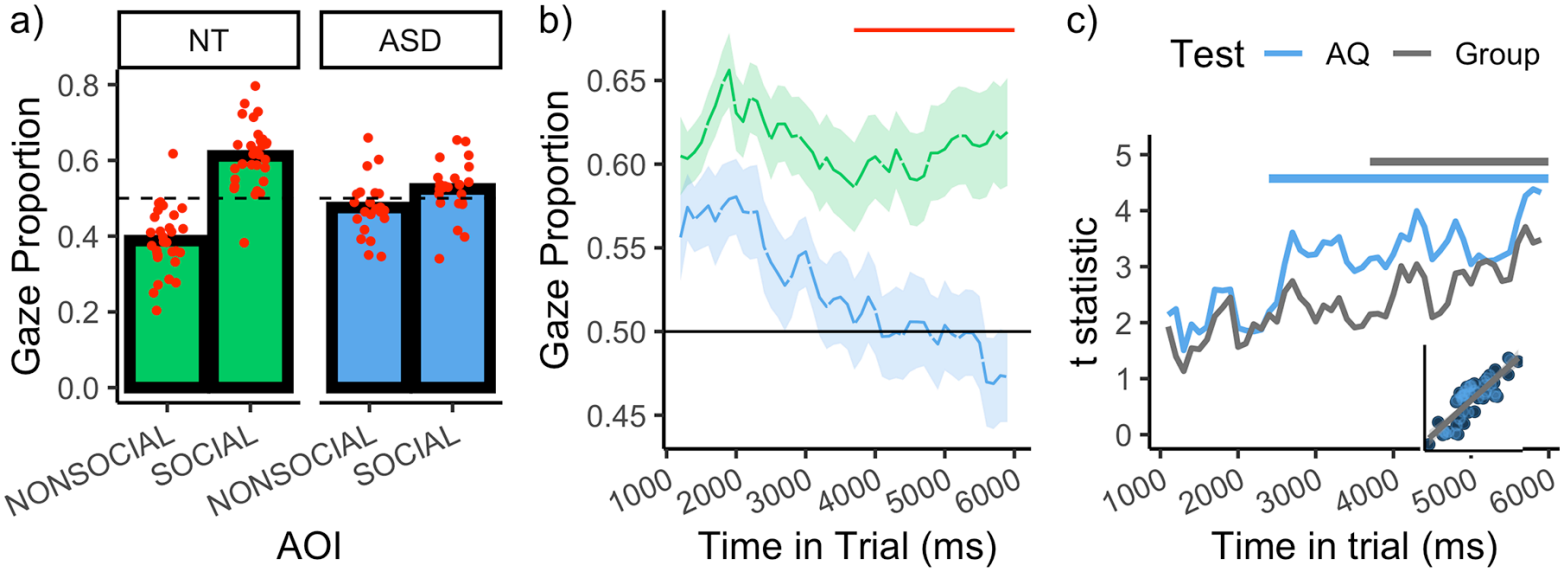
(a) Gaze proportion as a function of group and AOI. Red points indicate individual observer data. (b) Depicts gaze proportion to the social images over time as a function of group. Horizontal red line indicates the time bins wherein a difference between the groups was detected, according to a cluster-based analysis. (c) Lines depicts the *t* statistics for the test that gaze to the social image is predicted by AQ (blue) and Group (grey), as a function of time (the AQ test statistics were negative, predicting a bias towards the nonsocial image, but are plotted as absolute values to allow comparison with the Group test statistics). Note that we do not force AQ into a dichotomous variable – the *t* statistics reflect the outcome of a linear regression (i.e. the slope divided by the standard error). The horizontal lines demarcate the time bins wherein the corresponding statistic reaches the (cluster-corrected) threshold for reaching statistical significance. Inset plot shows the correlation between the t statistics for AQ and Group. All plots show the data for intact stimuli only.

The data were initially submitted to a general linear model with AOI (social, non-social), stimulus type (intact, scrambled) and Group (NT, ASD) as fixed effects. A main effect of AOI was detected, indicating a robust bias towards social images (*χ*^2^(1) = 24.84, *p* < 0.001). Critically, there was an interaction between AOI and Group, indicating a larger bias for social images in the NT group than the ASD group (*χ*^2^(1) = 17.29, *p* < 0.001). Moreover, the predicted interaction between AOI and stimulus type was detected *(χ*^2^(1) = 71.12, *p* < 0.001). The bias for social images was substantially larger in the intact condition (*β* = 0.15, *p* < 0.001) than scrambled condition (*β* = 0.02, *p* = 0.162). For the remaining analyses, only the data for intact images were tested (Figure 3(a)). The bias for social images was substantially larger in the NT group (*β* *=* 0.22, *p* *<* 0.001*)* than the ASD group (*β* *=* 0.05, *p* *=* 0.034).

### Temporal aspects of social bias

We next reduced each observer’s gaze data into the proportion of gaze within the social and non-social AOI in each 100 ms time bin from the start to end of the trial. We then removed data from the first 100 ms time bin, since it contained beyond 3 SDs less than the mean number of valid samples captured within all time bins. Figure 3(b), which depicts the proportion of gaze to the social AOI as a function of time and group, reveals several interesting aspects of gaze behaviour. First, there is a clear vertical offset between the NT and ASD functions, indicating a generalised increase in social attention in the NT group. However, the difference between the functions is not restricted to a simple vertical offset. In the NT group (upper line), the temporal profile appears to be characterised by three components (a) an initial increase social attention, (b) followed by a decline, and (c) then a partial ‘recovery’ towards the end of the trial. This profile is consistent with that found in a previous study of NT observers (Hedger et al., 2018). By contrast, this profile is not apparent in the ASD group (lower line), which instead resembles a simple linear ‘decay’ over time. It is also notable that the initial bias that ASD observers have for social images decays to the extent that it reverses at the end of the trial and a bias for *nonsocial* images is detected.

Figure 3(c) depicts the differences between the groups social gaze proportion over time, expressed as *t* statistics. This reveals a difference that increases over the course of the trial. Since conducting a statistical test for each time bin inflates the type 1 error rate, we protected against false-positives by applying a cluster-based permutation analysis (see Supplementary Material S2), which revealed that these group differences became robustly detectable in the final 2300 ms of the trial (3700–6000 ms, *p* = 0.005, red line in Figure 3(b)). Notably, as illustrated by Figure 3(b), the onset of this cluster corresponds closely with a clear inflection point where the NT function begins to recover, while the ASD function continues to decay.

We additionally framed the performance of our task in terms of its ability to distinguish between the two groups in a signal detection context (see Supplementary Material S2). The overall performance of the task (defined as area under the curve (AUC)) was comparable to previous eye-tracking tasks (AUC = 0.78 (0.65, 0.88), see Pierce et al., 2016) and analysing each time-bin independently revealed that maximum performance was obtained late in the trial, between 5600 and 5700 ms (AUC = 0.75, (0.62, 0.86)).

It is also informative (and potentially more powerful) to consider these variations in social attention over time at the trait level. Figure 3(c) depicts the extent to which observer’s AQ predicts gaze towards social images, expressed as a *t* statistic. These statistics increase over time and became statistically robust between 2400 and 6000 ms (*p* < 0.001). As revealed by Figure 3(c), the increase in these trait-level statistics over time mirror the increase in the group-level statistics over time, strengthening the conviction that the group differences genuinely relate to autistic traits. Notably, when conducting equivalent analyses with Age and IQ as predictors, we did not detect any evidence that these variables predicted a bias for social or nonsocial images at any point in time. This remained the case even when taking the least conservative approach and enhancing the power of these analyses by conducting no correction for the familywise error rate (Supplementary Material S2).

The above analyses converge on the idea that autistic differences in social attention increase after sustained viewing and become robust several seconds after stimulus onset, reflecting differences in the sustained maintenance of attention to social stimuli, as opposed to initial orienting.

### Model selection

**Figure 4.**
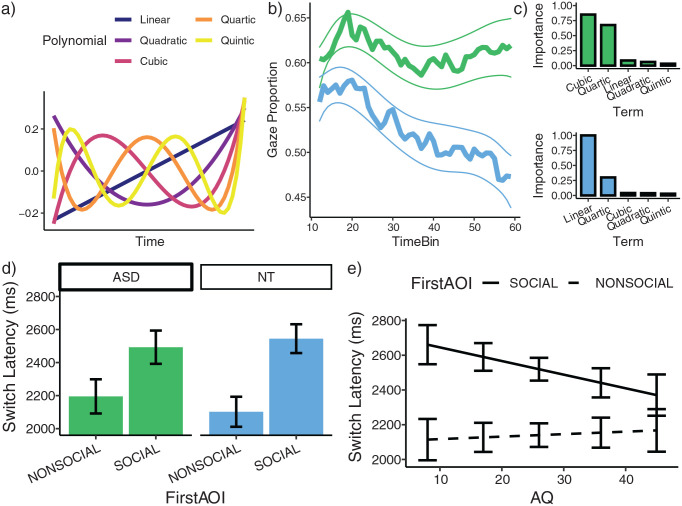
(a) Orthogonal polynomial timecodes (up to an order of 5). (b) Shows model-averaged predictions, wherein each models contribution to the prediction is weighted by it’s Akaike weight. Shaded region shows a prediction interval of *+/- 1SE*. (c) Upper: Shows the relative importance of each polynomial in explaining the NT gaze timeseries. Lower: Shows the relative importance of each polynomial in explaining the ASD timeseries. (d) Shows mean switch time as a function of group and the initially fixated image. (e) Shows mean switch time as a function of AQ and the image initially fixated.

To provide a parsimonious description of the divergent temporal profiles described above, we attempted to explain the time-series functions as a combination of orthogonal polynomials (Mirman et al., 2008) (Figure 4(a)). Our model selection procedure involved fitting all models with group (NT or ASD) and all combinations of these polynomials (up to an order of 5) as fixed effects, allowing for 2-way interactions (see Supplementary Material S3 for more details of the modelling approach). This implied fitting 40,069 models, whose performances were assessed according to the Bayes Information Criterion (BIC).

Subsequently, we used this set of models to generate model-averaged predictions, wherein the contribution of each model’s prediction to this averaging is weighted by it’s Akaike weight – entailing that more plausible models contributed more to the prediction (Wagenmakers & Farrell, 2004). These model-averaged predictions are shown in Figure 4(b) and seem to reproduce the major properties of the data well. The model-averaged parameter estimates can be found in supplementary material S3 (Table S1).

To examine the attentional dynamics underlying the gaze behaviour of each group, we determined the relative importance of each predictor for each group. To this end, we first re-fit all models to the NT and ASD data independently and summed the Akaike weights for each model in which each polynomial appeared. Intuitively, the sum of these values reflects the overall ‘support’ for the term across the population of models. These data are plotted in Figure 4(c). Inspection of this Figure reveals that the cubic and quartic terms are most important for describing the NT data, whereas the most important term in describing the ASD data was the linear term. Heuristically, the order of the polynomial can be thought of as reflecting the number of changes in the probability of fixating the social image (Mirman et al., 2008). We therefore interpret the relative importance of the linear term in the ASD group as indicating that the probability of fixating the social image tends to decay over time and does not tend to recover. By contrast, for the NT group, the increased support for the nonlinear terms is consistent with multiple successive stages of social attention. With reference to Figure 4(c), the general pattern is consistent with (a) an initial increase in social attention, (b) a decline, (c) a recovery and (d) the beginning of additional decay towards the very end of the trial, modelled by the quartic component.

One possibility is that the more linear decay of social attention observed in the ASD group reflects some generalised ‘sticky attention’ phenomenon that is not specific to social attention (Elsabbagh et al., 2013; Landry & Bryson, 2004). For example, it is possible that ASD observers may simply take longer to disengage attention from initially fixated images, regardless of their image category. Therefore, we next analysed the data in terms of switching behaviour. According to a generalised ‘sticky attention’ account, we would expect ASD individuals to exhibit later disengagement from an initially fixated image, regardless of whether it is social or nonsocial. Figure 4(d) depicts switch latency, as a function of group and the initially fixated image. Critically, we did not detect evidence for a generalised ‘sticky attention’ effect: there was no main effect of group on mean switch latency (*χ*^2^(1) = .006, *p* = 0.810.) Moreover, we detected no interaction between Group and the initially fixated image on mean switch latency (*χ*^2^(1) = .139, *p* = 0.240). However, when considering data at the level of individual traits, with AQ as the modelled explanatory variable, an interaction between AQ and initially fixated image on switch latency was detected (*χ*^2^(1) = 6.93, *p* = 0.008). Higher AQ was associated with faster switching from the social AOI, but not from the non-social AOI (Figure 4(e)). Thus, the data do not support a generalised slowing of disengagement, and instead indicate faster disengagement specific to social images in individuals with high autistic traits.

### Spatio-temporal similarity analysis

Beyond AOI-based analyses, we can refine our analyses further and directly quantify the extent to which observers gaze towards the same spatial locations of the experimental display over time. At the start of a trial, all observers will gaze at central fixation and so there will be little spatial deviation between individuals. As time progresses, we would expect this arrangement will be lost, and for the spatial deviation between individuals to increase. However, if the observer’s group membership (NT or ASD) meaningfully predicts distinct spatio-temporal patterns of gaze behaviour, we would expect this deviation to be greater between pairs of individuals from different groups than between pairs of individuals from the same group. To test this idea, we performed a spatio-temporal similarity analyses, akin to that recently employed in a genetic study of social attention (Kennedy et al., 2017).

**Figure 5.**
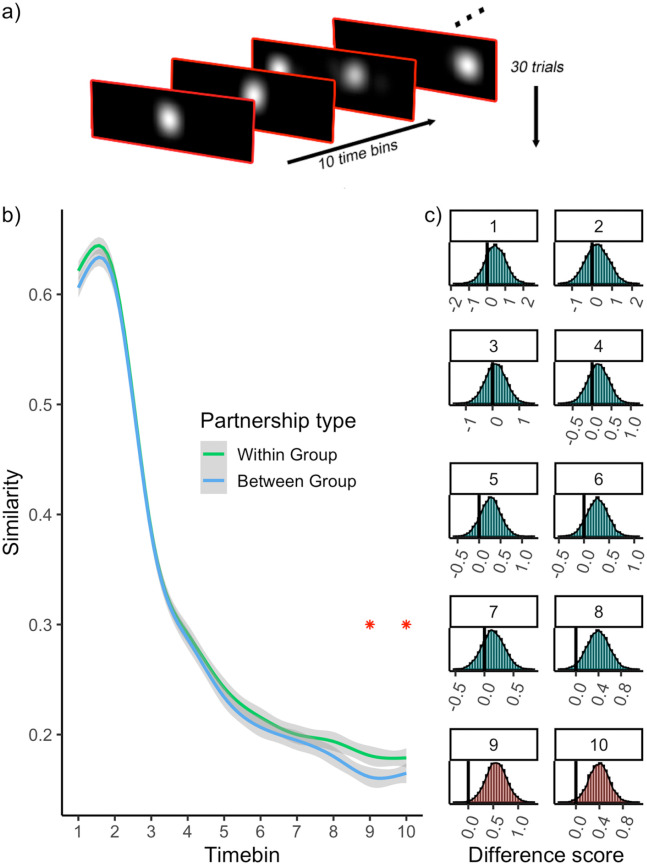
(a) Schematic of heatmap data for one observer. For each observer, heatmaps were created for each trial and timebin. (b) Similarity between heatmaps is plotted as a function of time and partnership type. Stars indicate the time bins wherein a difference is detected between within and between partnership types. (c) Histograms show the results of permutation tests for each time bin. The bars to the left of the vertical line demarcate the permuted difference scores that are more extreme than the empirically observed difference. Red histograms indicate the tests wherein a difference was detected.

We first created a spatial ‘heatmap’ of gaze position in each 500 ms time bin by defining coordinates corresponding to gaze location and multiplying these with a circularly symmetric Gaussian function (2 DVA at full width half maximum). This generated 30 (trials) × 10 (time bins) heatmaps for each observer (Figure 5(a)). For each heatmap, its similarity to the corresponding heatmap of every other observer (its *‘partner’*) was estimated by calculating the Pearson’s correlation between heatmaps. This yielded 397,800 values (30 trials × 10 time bins × 1326 unique *‘partnerships’*) that estimated the similarity of each pair of observer’s gaze behaviour in each trial and time bin. Of these partnerships, there were two ‘*partnership types’*. (a) *Within-group partnerships* (i.e. an ASD observer paired with another ASD observer, or an NT observer paired with another NT observer) and (b) *Between-group partnerships* (i.e. an ASD observer paired with an NT observer).

Figure 5(b) depicts the similarity between heatmaps as a function of time and partnership type. As expected, the general trend (independent of partnership type) is higher similarity at the start of the trial and a decline over time. The data also reveal enhanced similarity for within-group partners, particularly towards the end of the trial. If these differences are robust and reflect some meaningful distinction provided by the grouping of observers, we should expect them to be rarely obtained when random group labels are applied to the observers. We tested this possibility directly with a permutation test (Supplementary Material S4), which revealed that the group differences became statistically reliable within the final 2 time bins of the trial (5000–5500 ms: *p* = 0.024, 5500–6000 ms: *p* = 0.047) (Figure 5(c)).

## Discussion

In this study, we found evidence that individuals with ASD tend to exhibit a temporal profile of social attention that differs from NT observers. NT observers exhibited (a) a robust bias for social images after prolonged viewing (b) evidence for a ‘recovery’ of social attention after prolonged viewing. By contrast, observers with ASD exhibited (a) No detectable bias for either image category after prolonged viewing, with the gaze preference in the opposite direction to NT observers – tending towards *nonsocial* images (b) a stronger linear decay of social attention over time, with little evidence for ‘recovery’ late in the trial. Accordingly, divergence analysis revealed that the group differences in social attention increased over time, yielding a robust difference in the final ~2 s of the trial. These group differences were large in magnitude, robust and were closely related to individual variation in autistic traits. We observed similar group differences using spatiotemporal similarity analysis, where we found evidence that individuals within the same group were more likely to have similar gaze patterns than those from different groups, but only after prolonged viewing.

Our findings have several important implications. First, by considering the time-series components of the gaze data, we were able to capture the atypical viewing behaviour observed in ASD in a more granular way. We were able to determine, with precision, when group differences in social attention became detectable, make inferences about the number and onset of social attention changes over time and formally characterise these in a model-based way. Tracking these momentary changes in behaviour illustrates more vividly the underlying attentional trajectories that define how individuals with ASD deploy attention to social stimuli, placing further constraints on theories of diminished social attention in ASD.

Considering the temporal aspects of social attention provides insight beyond that gained from examination of global differences alone, because we can make informed inferences about the psychological basis of social attentional deficits during ‘early’ vs ‘late’ stages. Our data showed no differences in social attention in the early part of the trial, with ASD observers showing increasingly reduced social attention towards the later portions of the trial. Relevant to this finding, it is now reasonably well established that the initial few fixations after stimulus presentation are more stimulus-driven and heavily influenced by image salience (Johnson et al., 2015; Langton et al., 2008). Alternatively, it has also been proposed that initial orienting to social stimuli may be driven by specialised ‘quick and dirty’ subcortical mechanisms that rapidly prioritise conspecific stimuli in perceptual selection (Carlson et al., 2009; Tamietto & de Gelder, 2010). The temporal aspects of our analyses therefore afford us the specificity to speak against the notion that early, stimulus-driven attentional networks, or rapid conspecific detection pathways are impaired in ASD. This accords with recent lines of evidence from child and adult ASD samples indicating that the functioning of such mechanisms are within the normal range of NTs (Johnson, 2014).

It is also methodologically important that group differences were only staistically robust several seconds after stimulus presentation. This finding emphasises the importance of stimulus duration in similar paradigms designed to index social attention. For instance, it is possible that paradigms with short trial durations may be suboptimal to capture group differences in social attention. It also indicates that metrics that probe the initial selection of stimuli in the competition for attentional resources (e.g. latency to first fixation, proportion on first fixations, dot probe tasks) may not be optimally suited for revealing attentional differences between these populations. This idea is consistent with a relatively mixed evidence base produced by these measures, which paints a complex and inconsistent picture regarding whether initial orienting to social stimuli is genuinely reduced in ASD (Elsabbagh et al., 2013; Fischer et al., 2014; Greene et al., 2011; Moore et al., 2012).

What mechanisms could account for the increase in social attention differences over time? A well-established phenomenon from the eye-movement literature is the ‘gaze cascade’ effect (Shimojo et al., 2003), which holds that value-coding and gaze mutually interact, resulting in increased gaze towards preferred stimuli over time. We speculate that ASDs are associated with abnormal coding of social reward value, which disrupts a ‘gaze cascade’ type effect that maintains the perceptual selection of social stimuli over time in NT observers. Indeed, there is recent evidence to suggest that autistic traits are associated with reduced gaze-cascade effects (Hedger & Chakrabarti, 2021). This notion also fits well with data from a recent, novel paradigm that combined eye-tracking with a value-learning paradigm to investigate social attention in preschoolers (Wang et al., 2018). NT eye-movement behaviour was found to be consistent with increased value learning for social relative to nonsocial stimuli, whereas this pattern of behaviour was not observed in individuals with ASD. Thus, there is some support for the idea that reduced sensitivity to socially rewarding cues may underly the steeper decline in social attention and subsequent bias towards nonsocial images.

Regarding the novelty of our study, it is important to acknowledge that other studies have previously characterised temporal components of social attention in ASD. However, typically, this has been achieved via a less granular distinction between ‘early’ metrics such as latency to first fixation and ‘late’ metrics such as total gaze duration (Benson et al., 2009; van der Geest et al., 2002), or has involved a only a divergence analysis (Nakano et al., 2010; Sasson et al., 2007) to determine when group differences become detectable. Neither of these cases involve an explicit, model-based description of gaze as it evolves over time. We are aware of only one other, very recent study that employed similar model-based analyses of gaze behaviour in ASD (Del Bianco et al., 2020). In this study, it was observed that all observers showed an initial high probability of attending to faces in scenes, followed by a decline. Critically, NT participants were more likely to subsequently return gaze to faces than ASD participants – an effect that increased with age. Thus, our findings are in good agreement, and this convergent evidence strengthens our conviction that the divergent temporal profiles of social attention observed here are robust. Importantly, we demonstrate that such an effect translates to conditions with an explicitly competing rewarding nonsocial stimulus matched for low-level properties.

In interpreting our findings, several limitations need to be borne in mind. First, due to our desire for experimental control, our study involved competing presentations of carefully matched static images. This presents something of a contrast from natural viewing conditions, wherein competitions between social and nonsocial images do not tend to present themselves in such circumscribed ways – and tend to be more dynamic, diverse and unpredictable. As such, a plausible concern might be that group differences may be reduced due to a lack of ecological validity. However, it is notable that the sensitivity of our eye-tracking task (defined by AUC) exceeded that previously reported for video based paradigms such as the Geopref test (Pierce et al., 2011, 2016). Sensitivity aside, we must acknowledge that the behaviours we report from this paradigm are only a first approximation of how the relevant behaviours play out in the real world.

It is also important to note that the behaviours reported here from a cognitively able (but socially impaired) adult sample. Given the fact that a substantial proportion of individuals with ASD have some form of associated intellectual disability (Fombonne, 1999), we must therefore sensitive to the fact that we do not know whether these results will extend to participants with lower IQ, nor do we know what temporal profiles to expect in younger children with the condition. For this reason, we are enthusiastic about the prospect of applying this time-series approach to proximal stages of development, at ages during which symptomatology profiles are still emerging.

These interpretative cautions aside, our analyses demonstrate that considering the temporal structure of gaze signals can provide more refined quantitative endophenotypes for conditions marked by atypical social attention. Beyond the context of our observations, there are numerous examples from fields as diverse as ecology and cardiac medicine that demonstrate that important phenotypic differences are often more subtle and complex than simple mean shifts. Different cardiac health problems (Costa et al., 2005) and different species of worms (Brown et al., 2013), or fruitflies (Fulcher & Jones, 2017) can be classified by metrics such as multi-scale entropy, spectral flatness and temporal predictability/stationarity that describe how behaviour evolves over time. These insights, in combination with our own data, emphasise the importance of exploiting the time-varying nature of the signals under study as fully as possible, and inform future study design for paradigms measuring social attention.

## Supplemental Material

sj-pdf-1-aut-10.1177_1362361321998573 – Supplemental material for Autistic differences in the temporal dynamics of social attention Click here for additional data file. Supplemental material, sj-pdf-1-aut-10.1177_1362361321998573 for Autistic differences in the temporal dynamics of social attention by Nicholas Hedger and Bhismadev Chakrabarti in Autism

sj-pdf-2-aut-10.1177_1362361321998573 – Supplemental material for Autistic differences in the temporal dynamics of social attention Click here for additional data file. Supplemental material, sj-pdf-2-aut-10.1177_1362361321998573 for Autistic differences in the temporal dynamics of social attention by Nicholas Hedger and Bhismadev Chakrabarti in Autism

## Notes

1. We use the term ‘Individuals with Autism Spectrum Disorders’ for consistency, but we are aware that different sections of the autism community have preferences for either of these terms, and it is important to acknowledge these perspectives.

2. 2 NT observers had AQ scores that exceeded 26, and 1 ASD observer had a score of less than 26. The pattern of results was practically identical when these observers were excluded from the analysis.
