# Supplementary material for: Autistic differences in the temporal dynamics of social attention
Source: Autism. 2021 Mar 11;25(6):1615–26. doi: 10.1177/1362361321998573 (PMC8323332; doi:10.1177/1362361321998573)
Supplement: sj-pdf-1-aut-10.1177_1362361321998573 – Supplemental material for Autistic differences in the temporal dynamics of social attention [file sj-pdf-1-aut-10.1177_1362361321998573.pdf]

Supplementary Material

Article Title: Autistic Differences in the Temporal Dynamics of Social Attention

Journal Name: Autism

S1.

Selection and Matching of Image Pairs.

In our selection of stimuli, we excluded images that were i) low resolution ii) black and white iii) had a political connotation iv) involved famous people or events v) had sexually explicit content. Social images generally included pictures of happy individuals including couples, families, babies and children. Nonsocial images included rewarding content that did not include people, including food, scenery and money. A pilot sample of 23 participants (12 females) gave valence and arousal ratings for each image via a standard self-assessment manikin procedure (Bradley & Lang, 1994) . Observers made ratings on a 9 point likert scale, where a 5 rating conveys a neutral valence/arousal, and 6 or higher conveys a rewarding image or one that makes them feel ‘excited/jittery’.

All participants completed the valence ratings, but 2 males did not complete the arousal ratings. The Koch toolbox (Walther & Koch, 2006) was used to calculate image saliency (Itti & Koch, 2000) for each pair of images. The Koch Toolbox produces a composite measure of image saliency including features such as colour contrast and edge orientation calculated for each image. The global root mean squared (RMS) and mean luminance for each image was also calculated. To ensure that the image pairs presented on any given trial during the experiment were matched as closely as possible in dimensions other than their sociality, we chose the image pairings that minimised the difference between all affective (valence, arousal) and salience (RMS contrast, luminance, Koch Salience) metrics. No differences were detected between social and nonsocial image pairings in any metric.

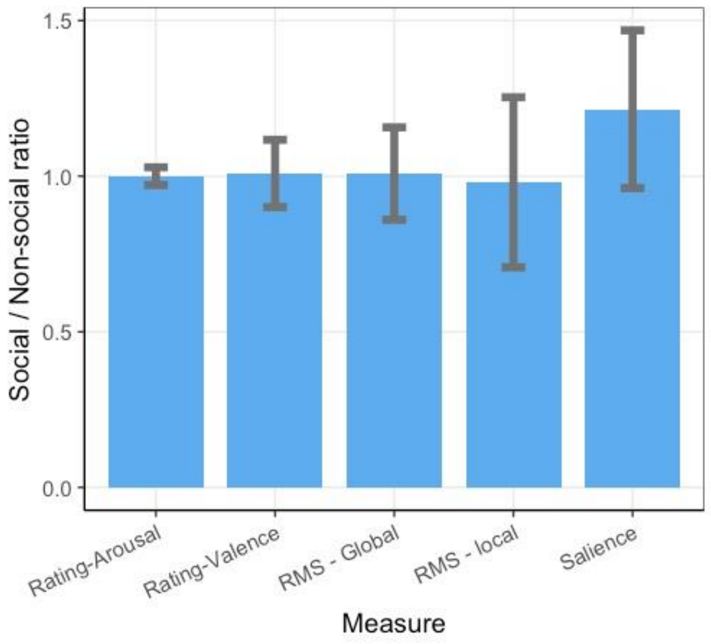

Figure S1. Comparisons of the social and nonsocial stimulus pairs including: arousal ratings; valence ratings; global root mean square (RMS) contrast; local RMS contrast; and stimulus saliency using Koch Toolbox. Error bars are 95% confidence intervals.

S2.

Cluster Definition and Permutation Analysis.

Our analyses revealed several time bins wherein an effect of a predictor on gaze behavior was detected. The rationale for grouping any such adjacent time bins into clusters is that one such time bin in isolation is more likely to reflect 'noise', whereas several adjacent time bins wherein the null hypothesis is rejected are more likely to reflect 'signal'. Therefore, 'clusters' of contiguous time bins are defined so that the size of the cluster can be incorporated into the correction for multiple comparisons and protect against type 1 errors (see below). Defining clusters in this way implicitly penalizes isolated time bins (more likely to be noise) relative to several adjacent time bins (more likely to be signal). Permutation analysis, described in detail in (Maris & Oostenveld, 2007) controls for familywise error rate associated with multiple

tests, whilst also taking into account the statistical dependencies in time-series data. Briefly, an initial test statistic is obtained for each time bin. Contiguous series of significant time bins are then defined (clusters). Within each cluster, the sum of statistics is obtained. Next, this process is repeated on 1000 randomly shuffled datasets, to obtain a null distribution of summed statistics. The p values reported in the main text therefore reflect the proportion of summed statistics in this null distribution that exceed that obtained from the empirical cluster.

ROC Analyses.

Figure S2A depicts the performance of the task in discriminating between individuals with and without ASD. The data are plotted as smoothed receiver operating characteristic (ROC) curves for each 100ms time bin. As with similar, previous studies (Pierce et al., 2016) , we defined performance of the task according to the area under the curve (AUC). The overall performance of the task (collapsed across time) was AUC = .78 [.65, .88]. Figure S2B depicts the performance of the task as a function of time. The peak sensitivity was obtained in the 5600-5700 time bin AUC = .76 [.62, .88].

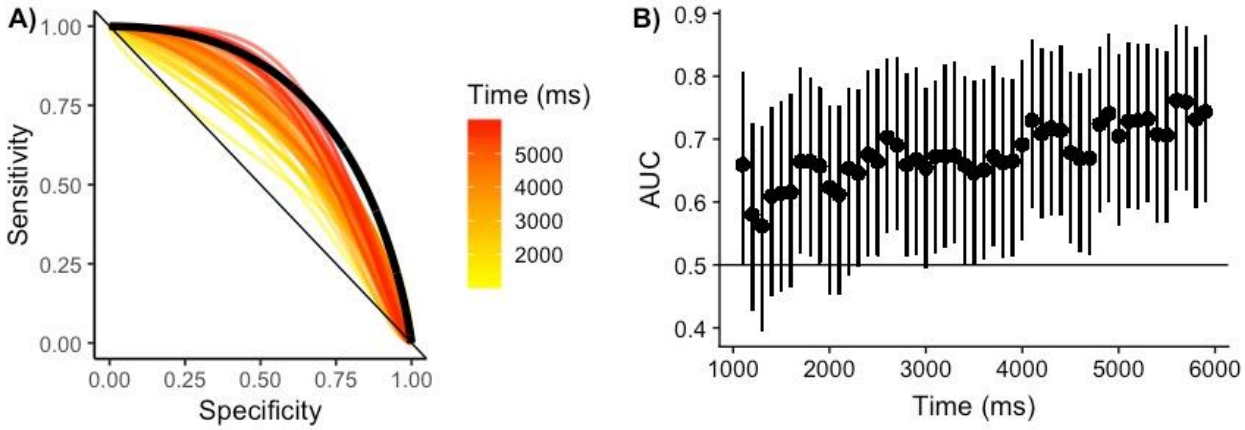

Figure S2. A) Smoothed ROC curves for each 100ms time bin of data. The curve for the entire task (collapsed across time) is shown by the thick black line. B) AUC as a function of time. Lines depict 95% confidence intervals obtained via bootstrapping.

Supplementary Divergence Analyses.

Additional analyses were performed to characterise the effects of IQ and Age in predicting bias towards social images over time. Visual inspection of Figure S3 reveals that neither of these statistics substantially deviated from zero across the duration of the trial: no effect was detected for either variable in any time bin, even if no correction for multiple comparisons was applied. Moreover, neither variable resembled the same linear increase observed over time observed for the Group and AQ statistics.

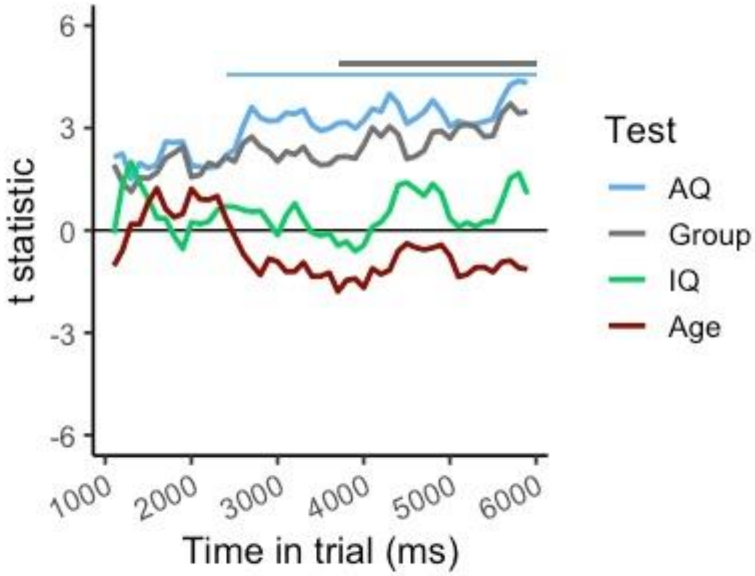

Figure S3. Depicts the t statistics for the test that each demographic variable predicts gaze towards the social AOI. Again, for the continuous variables (all except group), the t statistics reflect the outcome of a linear regression. The horizontal lines demarcate the time bins wherein the corresponding statistic reaches the (cluster-corrected) threshold for reaching statistical significance. Note that the IQ analysis only reflects the data for the ASD group (for which the IQ data were collected).

S3. Approach to modeling.

Given that our data resulted from an unconstrained free-viewing task, we made few a-priori assumptions about the nature of social attention changes over time. Orthogonal polynomials are particularly appropriate predictors in the context of our data since i) they can capture nonlinear relationships between gaze proportion and time ii) they are uncorrelated with one another, meaning that they capture distinct variance in the data and are therefore appropriate for multiple regression. However, given that there are an infinite number of orthogonal polynomials, this presented a potentially infinite number of candidate models that could be fitted to the data. Therefore, we placed a number of sensible restrictions on the models we tested.

As a general rule of thumb, the order of a polynomial can be thought of as reflecting the number of changes in probability in fixating the social image (a linear term is one change, a quadratic term is 2 changes - and so on). We reasoned that a five second trial is unlikely to be associated with more than 5 changes in probability and so restricted ourselves to polynomials up to an order of 5. Moreover, to enhance the interpretability of our models, we restricted ourselves to two-way interactions. Each fixed effect entered in the model was entered with a corresponding by-subject random slope.

Table S1.

Model-averaged parameter estimates

Parameter	β
Group	*-0.06*
Linear	-0.11
Quadratic	0.02
Cubic	0.05
Quartic	-0.57

Quintic	0.00
Group X Linear	-0.18
Group X Quadratic	0.01
Group X Cubic	-0.05
Group X Quartic	-0.02
Group X Quintic	-0.00

S4. Permutation Test

For each observer, we summed the similarity of all their within group partners and subtracted this from the summed similarity of all their between group partners. This yielded 53 difference scores, where positive values indicate greater similarity to within-group partners than between group partners. We then took the mean of these difference scores as our test statistic. To determine the chances of obtaining this statistic under random group membership, we performed the same analyses 10,000 times, each time randomly resampling the group labels without replacement. To obtain *p* values, we then calculated the number of these resampled statistics that exceeded the test statistic. Reported *p* values are adjusted according to the false discovery rate method.

References

Barr, D. J., Levy, R., Scheepers, C., & Tily, H. J. (2013). Random effects structure for confirmatory hypothesis testing: Keep it maximal. *Journal of Memory and Language*, *68*(3). https://doi.org/10.1016/j.jml.2012.11.001

Bradley, M. M., & Lang, P. J. (1994). Measuring emotion: The self-assessment manikin and the semantic differential. *Journal of Behavior Therapy and Experimental Psychiatry*, *25*(1), 49–59.

Itti, L., & Koch, C. (2000). A saliency-based search mechanism for overt and covert shifts of visual attention. *Vision Research*, *40*(10-12), 1489–1506.

Maris, E., & Oostenveld, R. (2007). Nonparametric statistical testing of EEG- and MEG-data. *Journal of Neuroscience Methods*, *164*(1), 177–190.

Pierce, K., Marinero, S., Hazin, R., McKenna, B., Barnes, C. C., & Malige, A. (2016). Eye Tracking Reveals Abnormal Visual Preference for Geometric Images as an Early Biomarker of an Autism Spectrum Disorder Subtype Associated With Increased Symptom Severity. *Biological Psychiatry*, *79*(8), 657–666.

Walther, D., & Koch, C. (2006). Modeling attention to salient proto-objects. *Neural Networks: The Official Journal of the International Neural Network Society*, *19*(9), 1395–1407.
